# Clinical decision support must be useful, functional is not enough: a qualitative study of computer-based clinical decision support in primary care

**DOI:** 10.1186/1472-6963-12-349

**Published:** 2012-10-08

**Authors:** Tiina Kortteisto, Jorma Komulainen, Marjukka Mäkelä, Ilkka Kunnamo, Minna Kaila

**Affiliations:** 1 School of Health Sciences, University of Tampere, Tampere, 33014, Finland; 2 The Finnish Medical Society Duodecim, PO Box 713, Helsinki, 00101, Finland; 3 Finnish Office for Health Technology Assessment at National Institute for Health and Welfare, PO Box 30, Helsinki, 00271, Finland; 4 Duodecim Medical Publications Ltd, PO Box 713, Helsinki, 00101, Finland; 5 The Hjelt Institute, University of Helsinki, PO Box 41, Helsinki, 00014, Finland

## Abstract

**Background:**

Health information technology, particularly electronic decision support systems, can reduce the existing gap between evidence-based knowledge and health care practice but professionals have to accept and use this information. Evidence is scant on which features influence the use of computer-based clinical decision support (eCDS) in primary care and how different professional groups experience it. Our aim was to describe specific reasons for using or not using eCDS among primary care professionals.

**Methods:**

The setting was a Finnish primary health care organization with 48 professionals receiving patient-specific guidance at the point of care. Multiple data (focus groups, questionnaire and spontaneous feedback) were analyzed using deductive content analysis and descriptive statistics.

**Results:**

The content of the guidance is a significant feature of the primary care professional’s intention to use eCDS. The decisive reason for using or not using the eCDS is its perceived usefulness. Functional characteristics such as speed and ease of use are important but alone these are not enough. Specific information technology, professional, patient and environment features can help or hinder the use.

**Conclusions:**

Primary care professionals have to perceive eCDS guidance useful for their work before they use it.

## Background

Health information technology (HIT), particularly electronic decision support systems [1,2], can reduce the existing gap between evidence-based knowledge and health care practice [3,4]. Benefits of computer-based clinical decision support (eCDS) to the quality of care and patient safety depend on the health care professionals’ acceptance and use of the eCDS in their decision making [5]. Automatic provision of eCDS in the workflow was found to be an independent predictor of improved clinical practice [6]. This means that eCDS guidance is provided automatically via electronic patient records (EPR) without extra effort by the professional [7]. Speed, real time delivery, and fitting in the workflow are important features for effective eCDS [8]. Too much guidance, e.g. drug interaction alerts, can be intrusive [9,10]. There is little evidence on eCDS in primary care or in settings where a variety of clinical areas need to be covered [11,12].

Health care professionals prefer patient-specific and relevant guidance, provided in a way that does not interfere with care or require inordinate effort and time [13,14]. In practice, plenty of factors influence the use of guidance [15]. Characteristics of the patient and the environment may be the most important factors for the acceptance of eCDS guidance [16]. Many barriers have been identified [17,18], particularly, too frequent or false alarms, lack of co-ordination between nurses and physicians, poor interface usability, time pressures, and inadequate training [19-22]. Facilitators include limiting the number of reminders, their ease of use and utility, documenting system problems and receiving feedback [21,23].

In Finland, electronic patient records and national guideline databases have long been used, which makes piloting a new HIT in primary care environment feasible [24]. Despite this, our implementation study of 18 months indicated that actual use of eCDS in the primary care context was only modest [25]. Therefore, we examine in this paper the professionals’ feedback more thoroughly. The main purpose was to increase our understanding for improving the application of the eCDS in the workflow of physicians, nurses, and other professionals [26]. The aim was to assess and describe in depth the specific reasons for using or not using the eCDS in primary care.

## Methods

### Study context

In one Finnish primary health care centre, patient-specific decision support (consisting of drug interaction alerts, reminders, and links to diagnosis-based guidelines) was shown automatically via EPR to physicians, nurses, and other professionals at the time of opening the record, entering a new diagnosis, or prescribing a drug (Table 1, see screen shots in additional files 1, 2,and 3). In addition, by selecting a specific function in the EPR, the physicians could see all reminders specific to the patient under their names on the relevant day’s appointment list (= virtual health check, VHC). (see description of the eCDS system [27] in additional file 4). Practical inclusion criteria for selecting the health centre were stable use of the EPR which included laboratory measurements, and recording of core patient information by the professionals, e.g. diagnoses and medications. The study protocol was approved by the ethics committee of the Pirkanmaa hospital district.

**Table 1:** **Computer**-**based clinical decision support** (**eCDS**) **functions** (**drug alerts**, **reminders**, **guideline links**, **and virtual health check** = **VHC**) **and procedures**: **examples**

**eCDS function**	**Procedure**	**An example**	
Drug alerts	In the prescription procedure, the eCDS system checks against the patient medication list whether the drug selected has an interaction with other medication and, if so, displays a pop-up with an interaction alert and a suggestion of drug change.	Warfarin, in the patient’s medication list, has an interaction with the medicine chosen (ibuprofen).	
		Do you want to choose another medicine?	
		Yes	No
Reminders	Patient-specific reminders are elicited by three trigger events in the EPR: 1) opening a patient record, 2) recording a new diagnosis, and 3) prescribing new medication.	*Short version*: Hypertension – check blood pressure?	
	*The short version* appears automatically, and *the long version* is shown when the mouse cursor hovers over the reminder.	*Long version*: This patient has hypertension. The latest BP measurement is more than a year old. Regular follow-up has been shown to improve the control of hypertension.	
Guideline links	*Guideline links* based on patient diagnosis-list and ICD-10 codes are shown when the user clicks the diagnosis.	*Guideline links*:	
		DG Hypertensio essentialis (primaria) I10	
		DG Fibrillatio atriorum I48	
		DG Angina pectoris I20	
Virtual health check	The user can run a VHC on her/his patients in the appointment schedule by clicking on the decision support file in her/his folder. Patient-specific decision support messages appear on the screen when the user clicks on *a specific link for a patient*.	Name: Matti Maa (PIC^a^): Control visit	
		*Reminders*	
		*Drug interactions*	
		*Contraindications*	
		*Guidelines*	
		Name: Anne Aamu (PIC^a^): Acute visit	
		*Reminders*	
		*Guidelines*	
		Name: Sanni Salo (PIC^a^): Diabetes control	
		*Reminders*	
		*Guidelines*	

^a^ PIC = personal identification code.

### Participants

We targeted the 48 professionals of the health centre; 15 physicians, 24 nurses (ward nurses, general practice nurses, and public health nurses) and nine other professionals (physiotherapists, head nurses, and a psychologist). All signed an informed consent form. During the study, four physicians left and four physicians entered the health centre.

### Data collection

Data collection took place between July 2009 and February 2011, by means of focus group interviews, a questionnaire, and spontaneous feedback.

#### Focus groups

In January 2010, six months after introduction of the eCDS to the primary care professionals, we convened three focus groups with the help of the chief officers. We aimed at involving as many physicians as possible, with at least one representative from preventive care, nurse practice, physiotherapy, and the two inpatient wards. Six physicians, five nurses, and one physiotherapist participated in one of two profession-specific groups or in one multidisciplinary group. The agenda was planned in advance by the moderator (TK) and facilitator (MK) and tailored to each group [28]. The aim was an open, informal discussion among participants about their experiences of the eCDS [29]. Broad themes such as general ideas about the eCDS, experiences of the use, practical problems, advantages /disadvantages for work, barriers to use and facilitators, and development issues guided the discussion. Each group discussion was audio recorded, transcribed, and the facilitator took notes on the general atmosphere and various pertinent issues [30].

In February 2011, at the end of the implementation period, a fourth focus group was convened for physicians. At this point, we wanted to gather opinions and experiences on the eCDS functionalities that were only available to physicians; 13 of the 15 physicians participated. The agenda was again planned ahead, using the previous broad themes, adding content and thresholds of reminders, by the moderator (JK) and facilitator (TK). JK acted as moderator, since his medical background was expected to facilitate discussion [31]. The aim was to obtain authentic feedback on the eCDS in physicians’ work. The group discussion was audio recorded and notes were made in a memo by a secretary.

#### Questionnaire

A questionnaire was sent to professionals in September 2010. After one reminder, 28 of the 45 professionals (62%) had responded; nine physicians, twelve nurses, two public health nurses, and five others. The six structured questions reported in this paper dealt with the system’s capacity and quality, as well as its perceived usefulness and ease of use, according to the technology acceptance model (TAM) [32]. It hypothesizes that perceived usefulness and ease of use are the main determinants of behavioural intention to use IT, which is expected to lead to actual use [33]. In this study, the perceived usefulness indicates the professional’s subjective probability for increasing her or his work performance by using the eCDS system. The perceived ease of use indicates the professional’s feelings for new system to be free of effort. In addition, reasons for using or not using the eCDS functions were queried in an open question: twelve out of 28 respondents (43%) elaborated their reasons.

#### Spontaneous feedback

The professionals were encouraged from the beginning to provide feedback via personal email, whenever they recognized specific issues or questions for system developers or researchers. The vendor added a feedback channel within the EPR-system from January 2010. Twelve spontaneous feedback messages were submitted by physicians; two via the personal email and ten via the EPR.

### Analyses

Two researchers (TK and MK) analyzed the qualitative data (focus group interviews, open question, and spontaneous feedback) by using deductive content analysis for testing previous theoretical issues in order to enhance understanding of the phenomenon [34]. The principle derived from the literature [35] was to identify features that helped or hindered the use of the eCDS, and how this was justified by the participants [36]. We first independently coded a categorization matrix consisting of the helping and hindering features. Next, we discussed the initial themes and grouped emerging themes by theory-based category (Table 2). The information in each category was condensed, reflected on, and interpreted jointly. Quantitative data were analyzed by using the software SPSS for Windows, version 15.0. The small sample size did not permit robust statistical analysis.

**Table 2:** **A theory**-**based framework of analysis with emerging sub**-**themes** – **helping** (+) **or hindering** (−), **quotations are described in text**


Content of computer-based decision support guidance	Usefulness for professional’s work (+)
	Non-helpfulness for professional’s work (−)
	Reliability (+)
	Quality (+)
System functionality	Ease of use (+)
	Speed (+)
	Too much, too small text (−)
	Impracticality (−)
Professional-associated features	Motivation (+/−)
	General attitude (+)
	Poor competence (−)
Patient-associated features	Reasons for visit (−)
	Low thresholds for reminders (−)
Environment-related features	Busy practice (−)
	Swine-flu epidemic (−)
Other features	Development issues (+)

## Results

In the end, the qualitative and quantitative analyses were combined. The main categories were the content of the eCDS guidance, the functionality of the eCDS system, the features related to the professions involved, the features of the patient groups, and environmental factors. A unique category of development issues emerged.

### The content of the eCDS guidance

The content of the guidance appeared to be a significant factor in the professionals’ decision to use eCDS in their work. Perceived usefulness was an essential reason for using eCDS in general or its particular function, e.g. drug alerts. Five out of nine physicians perceived the guidance as useful in their practice and as affecting their decisions, while one physician did not (Table 3). When asked about the usefulness of the eCDS, the majority of the nurses responded ‘I cannot say’. The nurses in preventive care, the physiotherapists, and the psychologist considered the eCDS guidance, mainly based on drug alerts, as unhelpful for their work. Reliability and quality were perceived as facilitating use (Table 3).

**Table 3:** **Perceived functionality and usefulness of the computer**-**based clinical decision support** – **number of responses in the questionnaire**

		**Physicians**	**Nurses**	**Others**	**Total**
		***n*= 9**	***n*= 13**	***n*= 5**	***n*= 27**
It is easy to use	Yes	7	4	3	14
	No	0	1	0	1
	Cannot say	2	8	2	12
It is rapid enough	Yes	6	6	4	16
	No	1	0	0	1
	Cannot say	2	7	1	10
It is reliable	Yes	7	7	2	16
	No	0	0	0	0
	Cannot say	2	5	2	9
It is of high quality	Yes	5	3	2	10
	No	1	0	0	1
	Cannot say	3	9	3	15
It supports my work	Yes	5	3	0	8
	No	1	3	1	5
	Cannot say	3	6	3	12
It influences my decisions	Yes	5	1	1	7
	No	1	4	3	8
	Cannot say	2	7	1	10

The physicians’ consensus was that the guideline links and VHC were not useful; the guideline texts were too long, and use of the VHC function would have necessitated more time for paper work. Only physiotherapists considered the guideline links useful.

From the physicians:

"‘Good system; it does not disturb, some reminders are useful.’"

"‘Interaction alerts with a prescription are useful.’"

"‘Drug alerts motivate to clear up medication lists, but it takes time.’"

"‘I think that there is too much text … too many links.’"

"‘Use of virtual health checks requires more time for paperwork.’"

From the other professionals:

"‘Guideline links are useful for the physiotherapist.’"

"‘For our work [health promotion] and customer relationships, there aren’t necessarily relevant things.’"

"‘Mainly drug interaction alerts, and I [nurse] do not prescribe.’"

"‘Okay, good information but it does not influence my work much in practice, how you use it depends on the group of professionals you belong to.’"

"‘Most of the drug alerts are not relevant for physiotherapists’ work.’"

"‘Reminders do not support psychologist’s work’"

### The functionality of the eCDS system

The majority of the physicians and other professionals found the ease of use and speed good, while most of the nurses expressed no opinion, stating that they could not answer these questions (Table 3). The physicians reported that specific features of the system (EPR, eCDS, or their integration) were hindering the use. These included known problems with the EPR system, reminders’ position on the screen, irritating drug interaction and contraindication alerts, and a lack of structured recording of smoking status (causing inappropriate reminders). The nurses experienced the eCDS as impractical for work at a busy call centre.

From the physicians:

"‘There was generally a problem with the functioning of the electronic patient record system … yes, big problems with the computer.’"

"‘Reminders’ position on the left side of the screen.’"

"‘If a patient has 20 reminders, I just go past them quickly.’"

"‘Reminders’ texts are sometimes too strict in the short version. If you don’t move the cursor over the text and see the whole reminder, the wording doesn’t work.’"

"‘Too low triggering threshold with drug interaction alerts. I never bother to read them.’"

"‘Irritating alerts.’"

"‘Excess alerts – e.g., asthma and opiate, warfarin and paracetamol.’"

"‘The reminder “great cardiovascular risk [SCORE], clarify the patient’s smoking status…”. It is not possible to record smoking status in a structured manner in the EPR.’"

From the nurses:

"‘We [general-practice nurses] receive such a staggering number of phone calls that we really don’t look for anything, just do exactly what they [patients] call about.’"

### The features related to professionals

The health care professionals’ motivation to learn to use a new IT system in practice seemed to vary. Some nurses discussed inadequate skills or interest in using IT in general. Primary care professionals seemed to have generally positive attitudes toward future use of eCDS, but more training and time to learn to use a new system were described as necessary. In addition, it was suggested that a local opinion leader could be useful to teach others.

From the physicians:

"‘I think that we should use it more.’"

"‘Do not need. I use Terveysportti [national health portal for professionals].’"

"‘I get an exact search when I use Terveysportti.’"

"‘Should there be an enthusiast or someone who uses it and thinks about it, and then tells us what the tricks are, what is good?’"

From the nurses:

"‘We have certainly more potential than we have been used to … Yes, it is absolutely a good thing … Yeah, I would say the same, learn to use it and intensify its use then’"

"‘All experienced this training as necessary.’"

"‘The individual differences, where you are used to searching for information… e.g., poor IT skills or no interest in the computer.’"

### Features of the patient groups

The patient’s agenda for the visits was the most important issue for the physicians. It was common that the eCDS guidance was ignored because it did not address the reasons for the encounter. The physicians’ spontaneous feedback indicated another factor, in overly sensitive patient-specific triggering cut points and irrelevant reminders.

"‘The patients usually have a reason for their visit. This has to be the primary focus for the physician. If there are ten VHC reminders even before the patient arrives, there is in no way enough time.’"

"‘My patient had a fasting plasma glucose value of 6.0 once, previously normal. Decision support suggests diabetes. To my mind, a single FPG value of 6.0 does not justify diagnosing diabetes.’"

### Environmental factors

An unforeseeable external factor arose during the study, the swine-flu epidemic with concomitant large-scale vaccination of the population, resulting in changes to at least nursing practice for many months and clearly causing extra work. A common barrier to use was busy practice in primary care. In particular, nurses reported that changes in their practice would have been needed before they could use the system.

"‘The swine-flu epidemic has consumed an awful lot [of time], we didn’t even have any general-nurse practice appointments available for a long time.’"

"‘When I am busy, I don’t look for anything really.’"

"‘Nothing more than simply doing what I have to do.’"

"‘In that situation, I could imagine that once you get acquainted with one [specific disease], it will be more useful than it is now.’"

### Development issues

The physicians hoped that the interface design would become more visually oriented. They also proposed some new alerts or eCDS functions that could better assist in their work or make the eCDS system consistent.

"‘Make the system more visual; nice and desirable like an iPad… to give the user the feeling “Oh, what else could I look for? I want more of this.”’"

"‘If it were possible, a visual sign like when Microsoft™ e-mail shows pop-ups for new mail.’"

"‘A transient box, in the middle enough, for a short enough time, and automatically [closing]. If there would be a movement, then it would be noticed. […] it should be neutral on the screen and definitely should not disturb.’"

"‘Request for a new interaction alert: enoxaparin or dalteparin prescription when a patient has warfarin medication. This alert reminds physician to check the appropriateness of continued simultaneous use.’"

"‘Patient-tailored threshold values, different from guideline-based’"

"‘In the virtual health check retrospective testing, one slim patient got an obesity warning, because of a flawed weight or height recording. It was related to a missing digit, a recording of the height as 52 cm instead of the correct 152 cm. These types of typos should be guarded against by the system.’"

## Discussion

The main findings of our study are twofold: perceived usefulness results in professionals using eCDS guidance while perceived non-helpfulness leads to non-use in primary health care. The guidance has to be designed for each profession and tailored, so that also e.g. nurses and physiotherapists can find it relevant for their practice. The profession groups have their own duties and specific practices; therefore their information needs vary greatly [37-40]. Our study indicates that even within one profession there are differences in the perceived usefulness of eCDS guidance. Lugtenberg et al. [41] reported similarly that perceived barriers for Dutch general practitioners varied greatly with the set of guideline recommendations involved.

Our results are in accordance with Davis’s technology acceptance theory [32], wherein perceived usefulness is considered a determinant with the main determinants of attitude and behavioural intention to use IT. Our results are strengthened by our use of multiple study methods: both open and structured questions, as well as focus group discussions with participants describing their specific reasons for using or not using the eCDS system or guidance. According to McDermott [14], perceived usefulness is a key characteristic in acceptance of a computer-based intervention among physicians. We discovered in addition that, while the majority of the physicians perceived the eCDS guidance to be useful for their work, the opposite was true for nurses and others, who sometimes appeared unable even to answer the question.

It is interesting that perceived usefulness showed in the data more consistently than perceived ease of use. It may be that the eCDS system basically was easy to use, since it had been integrated to the EPR system and the guidance popped on screen without specific effort, indicating perceived ease of use in TAM model [32]. Seven of nine physicians replied ‘yes’ to the question of easy to use, and the one negative answer came from a nurse (Table 3). It is conceivable that perceived ease of use, together with the main determinants of attitude and behavioural intention to use, was influencing in the background to the professionals’ perceived usefulness of the eCDS as TAM suggests [32].

The functionality of the eCDS system seemed good. Most responded positively to the questions dealing with this, with only one physician and nurse answering in the negative. However, the majority of the nurses found themselves unable to answer these functionality questions, which seemingly confirms the failure of the implementation among nurses [25]. In general, the professionals did not report any eCDS-system-based problem encumbering their work. By contrast, they seemed satisfied, because the eCDS system did not disturb them or force them to do anything. Indeed, this has been cited elsewhere as a key feature of a successful eCDS system [13]. According to the physicians, a more visually oriented system might be more desirable to them.

The results reaffirm that plenty of barriers exist to the use of reminders, even automatic ones, in primary care [17,19-22]. These were related to the studied eCDS system, inaccurate data in the EPR or code mismatch between the EPR and the eCDS system, the usability of the EPR itself, the groups of professionals, and patient preferences. The list bears a remarkable resemblance to the list of hurdles for use of clinical information systems as well as clinical guidelines [15,42]. One exception was seen: lack of agreement with the recommendations was evaluated as the most common perceived barrier to use of guideline recommendations among Dutch physicians [43], while this was not brought up as a barrier in our study. On the contrary, all respondents reported reliance on the reliability of the eCDS guidance.

The swine-flu epidemic and full-scale vaccination effort caused clear and measurable changes, especially in the nurses’ practice, for several months. It probably had a negative influence on the eCDS implementation and use, as busy practice was a common environmental reason for non-use of the eCDS. A recent usability evaluation study of a CDS tool for osteoporosis disease management [44] also indicated that physicians’ lack of time was a major challenge to point-of-care use of the tool.

A facilitating factor seems to be a generally positive attitude toward eCDS among primary care professionals. They did report needing more training and more time to learn to use the eCDS system. In particular, nurses expected some changes to their general nursing practice before actual implementation of the eCDS. Indeed, good training has been seen to be associated with favourable assessment of IT among nurses [45].

### Strengths and limitations

Generalization of qualitative results can be problematic by the standards of the positivist approaches typical of quantitative research, in view of issues such as the representativeness of the sample [31]. Generalization can, however, be approached from a theoretical standpoint that takes into account the nature of the study question and the framework. So we can postulate that our results strengthen existing knowledge of the meaningful elements behind eCDS use among health care professionals. By using multiple methods and a long time period, we were able to extend our understanding of primary care realities during the implementation phase [46]. We believe that these results have validity for application in other primary care settings where a new automatic eCDS system integrated with EPR is being introduced.

Malterud [46] raises another issue, surrounding the researchers’ commitment to reflexivity. Our preconceptions stemming from previous studies [47,48] directed our initial choices and influenced the study process. We therefore chose to use deductive methods in the qualitative analyses and to describe the process as explicitly as possible. Four of us have a background in medicine and one in physiotherapy, which might aid us in understanding the different perspectives of the professionals. Two of us (JK and IK) also participated in development of the target eCDS system, which could have affected the study. From the beginning of the eCDS development, a study project was in progress to describe and evaluate what was going on in practice when the eCDS was piloted [49,50] and implemented [25]. The same choice has been made in many previous studies that involve IT developers as researchers [5,51].

A limitation is our use of a non-standardized questionnaire. However, the questions reported here were planned based on the theory [32] and the evidence of applied research [52,53]. Limited sample size and potential selection bias in responses due to participated versus non-participated professionals decrease a generalization of the results.

## Conclusions

Perceived usefulness seems to be decisive for the use of eCDS guidance in primary care practice, and therefore the content of the eCDS is a critical issue. Information needs of profession groups in various environments (e.g., preventive care) have to be determined, and the eCDS guidance tailored according to those needs. Functionality-related characteristics such as speed and ease of use of the eCDS system and reliability of guidance are important but not sufficient in relation to uptake by primary care professionals.

Although this study targeted multi-profession groups in primary care, the studied eCDS system had been essentially based on medical evidence and to aid in physicians’ work. In this respect, there are several issues for future studies, e.g. targeting to patient’s reason for visit and tailoring with patient-specific threshold values. Unless future research considers in depth all professions’ needs, experiences and opinions the full potential of eCDS services may not become a reality.

## Competing interests

Authors TK and MM declare that they have no competing interests. JK is Editor-in-chief of Current Care Guidelines, Finnish Medical Society Duodecim, and a member of the editorial board for EBMeDS, Duodecim Medical Publications Ltd. IK is a salaried employee of Duodecim Medical Publications Ltd, the company that develops and licenses the EBMeDS decision support service. MK chairs the Current Care Guidelines board at Finnish Medical Society Duodecim.

## Authors’ contributions

All authors were involved in conceiving the study and designing the questionnaire and interview forms. TK was responsible for data collection and analysis. TK led the writing process, supervised by MK, and all authors commented on sequential drafts and approved the final version of the manuscript.

## Pre-publication history

The pre-publication history for this paper can be accessed here:

http://www.biomedcentral.com/1472-6963/12/349/prepub

## Supplementary Material

Additional file 1 Patient specific automatic reminders and guideline links on the electronic patient record screen: a patient with diabetes as an example. Click here for file

Additional file 2 Patient specific drug interaction alert function on the electronic patient record screen. Click here for file

Additional file 3 Virtual health check results within the physician’s appointment schedule. Click here for file

Additional file 4 Computer-based Clinical Decision Support: An overview of the Evidence-Based Medicine electronic Decision Support (EBMeDS) architecture and decision support rules (= scripts). Click here for file
