# Supplementary material for: Clinical decision support must be useful, functional is not enough: a qualitative study of computer-based clinical decision support in primary care
Source: BMC Health Serv Res. 2012 Oct 8;12:349. doi: 10.1186/1472-6963-12-349 (PMC3508894; doi:10.1186/1472-6963-12-349)
Supplement: Additional file 1 — Patient specific automatic reminders and guideline links on the electronic patient record screen: a patient with diabetes as an example. [file 1472-6963-12-349-S1.pdf]

Additional file 2a Patient specific automatic reminders and guideline links on the electronic patient record screen: a patient with diabetes as an example

There are three trigger events in the electronic patient record that elicit or update patient-specific clinical decision support on the left side of the screen:

- opening the patient record
- recording a new diagnosis
- prescribing a new medication

Case: A patient with diabetes (Name: EBMEDS DIABEETIKKO, ID: 010160), for whom four reminders and eleven diagnosis based guideline links (below) were automatically triggered when the physician opened the patient record.

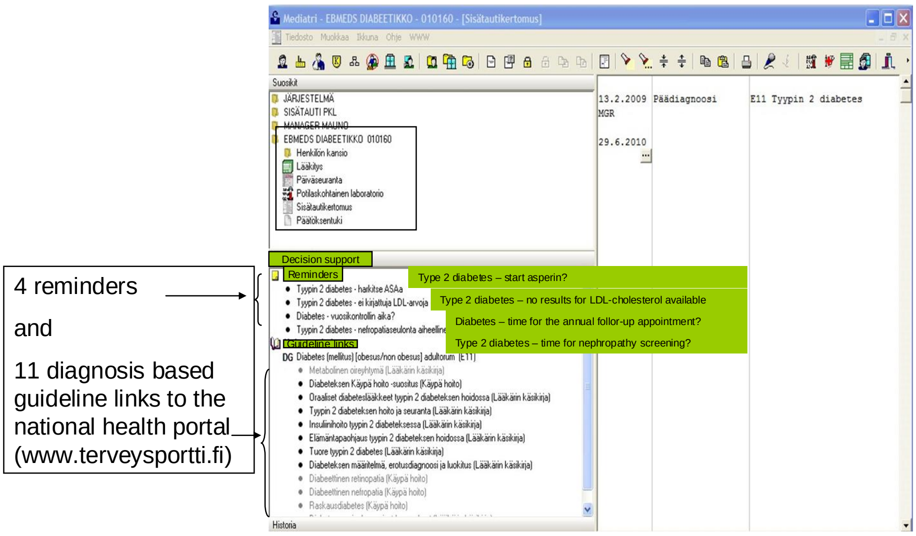

A short version of the reminders is shown automatically. When the mouse cursor is placed over the reminder, a long version appears on the screen. If the user is a nurse, additional information relevant for nurses is shown in the reminder.
