# Supplementary material for: Clinical decision support must be useful, functional is not enough: a qualitative study of computer-based clinical decision support in primary care
Source: BMC Health Serv Res. 2012 Oct 8;12:349. doi: 10.1186/1472-6963-12-349 (PMC3508894; doi:10.1186/1472-6963-12-349)

Additional file 2b Patient specific drug interaction alert function on the electronic patient record screen

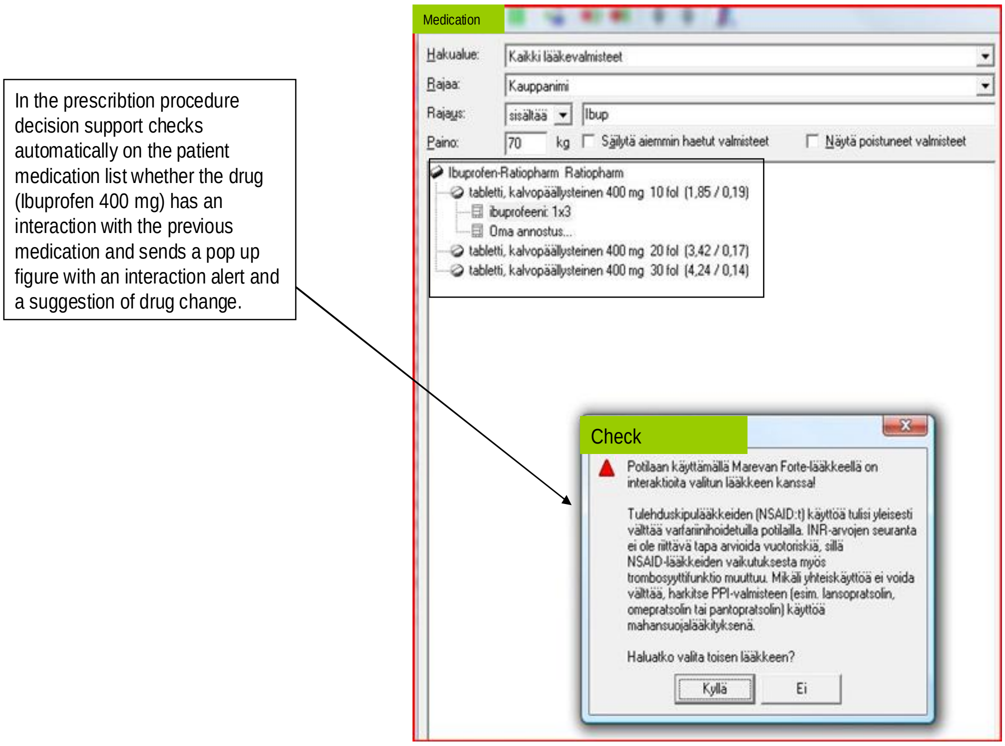

Supplement: Additional file 2 — Patient specific drug interaction alert function on the electronic patient record screen. [file 1472-6963-12-349-S2.pdf]
