# Supplementary material for: Clinical decision support must be useful, functional is not enough: a qualitative study of computer-based clinical decision support in primary care
Source: BMC Health Serv Res. 2012 Oct 8;12:349. doi: 10.1186/1472-6963-12-349 (PMC3508894; doi:10.1186/1472-6963-12-349)
Supplement: Additional file 3 — Virtual health check results within the physician’s appointment schedule. [file 1472-6963-12-349-S3.pdf]

Additional file 2c Virtual health check results within the physician’s appointment schedule

User (Name: MANAGER MAUNO) can run a virtual health check (VHC) on her/his patients in the appointment schedule. This happens when the user clicks on the decision support document in her/his folder (step 1). All patient-specific decision support messages appear on the right side of the screen (step 2). Patient specific reminders (short versions), drug alerts and guideline links appear when the user clicks on the specific CDS link (steps 3).

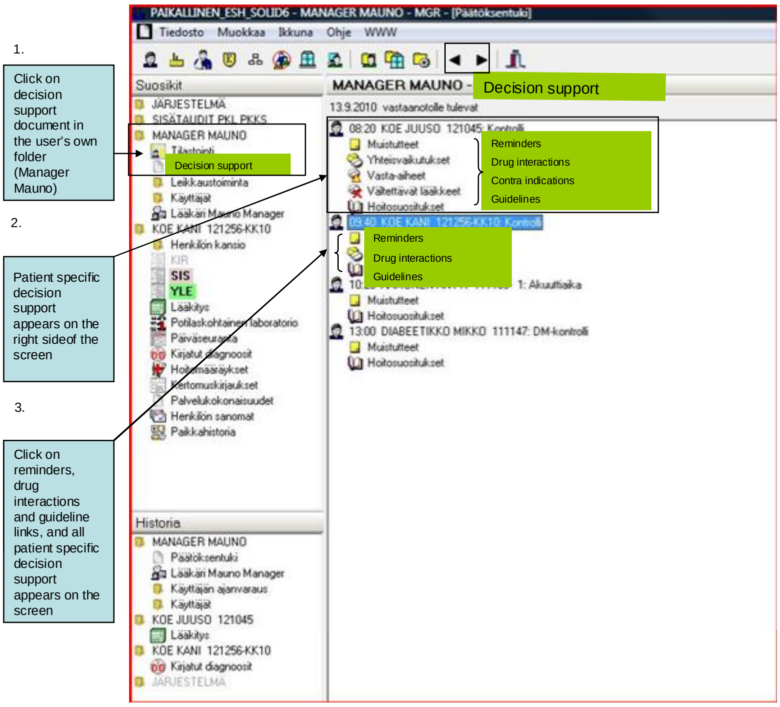

In the next figure all the patient specific (Name: KOE KANI) reminders, interaction alerts and diagnosis-based guideline links are shown. The long versions of the reminders with additional sentences for nurses can be seen when the mouse cursor is placed over the reminder (step 4).

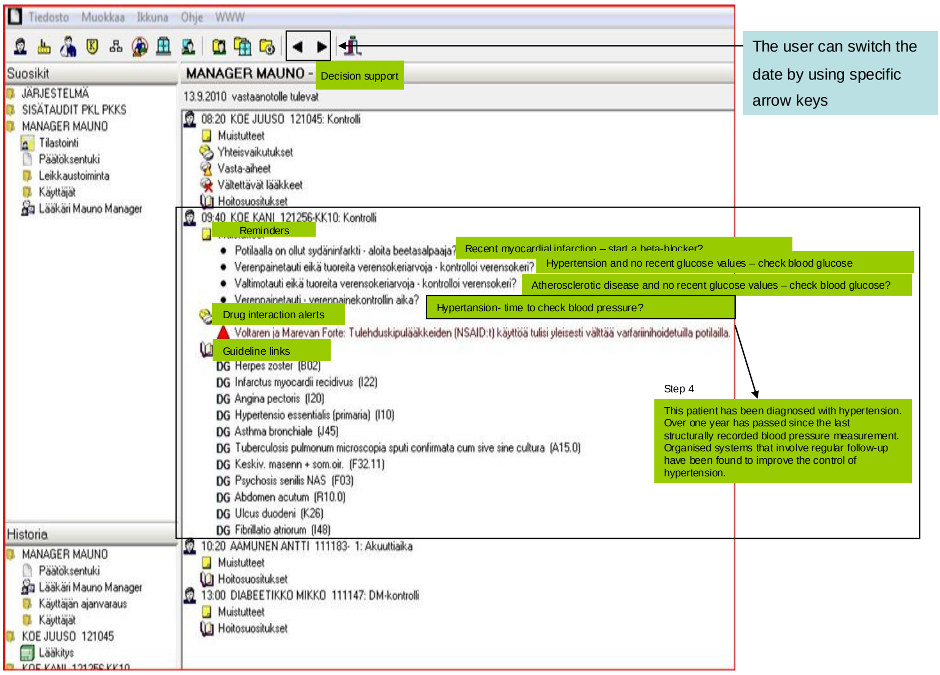

Tiedosto Muokkaa Ikkuna Ohje WWW
The user can switch the date by using specific arrow keys
Suosikit
MANAGER MAUNO -
Decision support
13.9.2010 vastaanotolle tulevat
08:20 KOE JUUSO 121045: Kontrolli
Muistutteet
Yhteisvaikutukset
Vasta-aiheet
Vältettävät lääkkeet
Hoitosuositukset
09:40 KOE KANI 121256-KK10: Kontrolli
Reminders
Potilaalla on ollut sydäninfarkti - aloita beetasalpaaja?
Recent myocardial infarction – start a beta-blocker?
Verenpainetauti eikä tuoreita verensokeriarvoja - kontrolloi verensokeri?
Hypertension and no recent glucose values – check blood glucose
Valtimotauti eikä tuoreita verensokeriarvoja - kontrolloi verensokeri?
Atherosclerotic disease and no recent glucose values – check blood glucose?
Hypertansion- time to check blood pressure?
Drug interaction alerts
Voltaren ja Marevan Forte: Tulehduskipulääkkeiden (NSAID:t) käyttöä tulisi yleisesti välttää varfariinihoidetuilla potilailla.
Guideline links
DG Herpes zoster (B02)
DG Infarctus myocardii recidivus (I22)
DG Angina pectoris (I20)
DG Hypertensio essentialis (primaria) (I10)
DG Asthma bronchiale (J45)
DG Tuberculosis pulmonum microscopia sputi confirmata cum sive sine cultura (A15.0)
DG Keskiv. masenn + som.oir. (F32.11)
DG Psychosis senilis NAS (F03)
DG Abdomen acutum (R10.0)
DG Ulcus duodeni (K26)
DG Fibrillatio atriorum (I48)
Step 4
This patient has been diagnosed with hypertension. Over one year has passed since the last structurally recorded blood pressure measurement. Organised systems that involve regular follow-up have been found to improve the control of hypertension.
Historia
10:20 AAMUNEN ANTTI 111183- 1: Akuuttiaika
13:00 DIABEETIKKO MIKKO 111147: DM-kontrolli
