# Supplementary material for: Clinical decision support must be useful, functional is not enough: a qualitative study of computer-based clinical decision support in primary care
Source: BMC Health Serv Res. 2012 Oct 8;12:349. doi: 10.1186/1472-6963-12-349 (PMC3508894; doi:10.1186/1472-6963-12-349)
Supplement: Additional file 4 — Computer-based Clinical Decision Support: An overview of the Evidence-Based Medicine electronic Decision Support (EBMeDS) architecture and decision support rules (= scripts). [file 1472-6963-12-349-S4.pdf]

Additional file 1 Computer-based Clinical Decision Support: An overview of the Evidence-Based Medicine electronic Decision Support (EBMeDS) architecture and decision support rules (= scripts)

“The Evidence-Based Medicine electronic Decision Support (EBMeDS) system brings evidence into practice by means of context-sensitive guidance at the point of care. EBMeDS receives structured patient data from electronic patient records (EPRs) and returns reminders, therapeutic suggestions and diagnosis-specific links to guidelines. It can also be used to canalize patient data to electronic forms and calculators. In addition to real-time use, the EBMeDS decision support rules can also be run in patient populations ("virtual health checks").” (www.ebmeds.org)

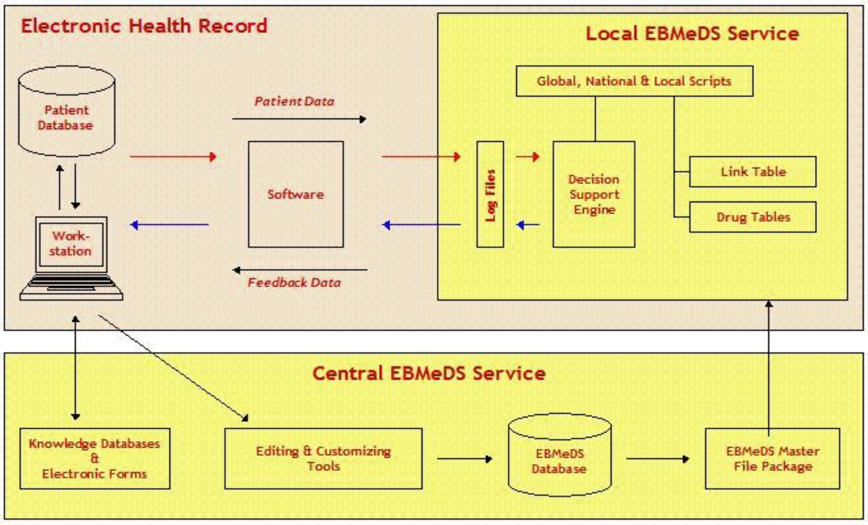

“The EBMeDS module can be integrated into any electronic patient record system (EPR) capable of providing structured data. The EPR sends patient data as an XML message from/to the EBMeDS module. The module analyzes the data package and returns an XML message containing the decision support information. The EPR vendor designs the user interface, in which the decision support information is shown in the EPR work station.

Decision support rules can be created and edited with a web-based editor, which is available for all end users. The editor works against a database, from which the production package for the EPRs is compiled.
Descriptions of published decision support functions are available free in the Script Description Database on the web pages. The documents contain a plain language summary describing the functionality in a few words, links to the evidence (by subscription; including Cochrane reviews, evidence summaries from EBM Guidelines and references), and the script messages. Potential harms are described separately.

Example of a plain language summary

If a patient with premenstrual syndrome visits repeatedly (at least twice within 2 years) and is not using SSRIs (or other antidepressants), a reminder on the potential benefit of SSRIs is shown. The reminder will disappear when more than 3 months have passed from the last diagnosis. For a patient with premenstrual syndrome, a reminder is shown suggesting SSRIs if the symptoms are severe.

Several structured databases are being constructed by Duodecim and other organizations to aid decisions in drug therapy . These databases include indications, contraindications and drug interactions. ” (www.ebmeds.org)

More information at, www.ebmeds.org.
